# Identification of Child Abuse and Neglect Based on the Perception of Training Physicians in Jeddah, Saudi Arabia

**DOI:** 10.7759/cureus.56985

**Published:** 2024-03-26

**Authors:** Reem Alyoubi, Majed M Al-Hayani, Ahlam Mazi, Alkhansaa O Bajawi, Anas S Alyazidi, Summayah A Kobeisy

**Affiliations:** 1 Department of Pediatrics, King Abdulaziz University, Jeddah, SAU; 2 College of Medicine, King Abdulaziz University, Rabigh, SAU; 3 College of Medicine, Fakeeh College for Medical Sciences, Jeddah, SAU; 4 College of Medicine, King Abdulaziz University, Jeddah, SAU; 5 Department of Pediatrics, Dr. Soliman Fakeeh Hospital, Jeddah, SAU

**Keywords:** practicing physicians, medical education, saudi arabia, child neglect, child abuse

## Abstract

Introduction

Child abuse and neglect (CAN) affects many countries, including Saudi Arabia (SA). CAN in SA is more commonly detected in hospitals. Therefore, healthcare professionals must identify and report the cases. This study aims to assess knowledge and perceptions toward CAN among training physicians.

Methodology

A cross-sectional survey was conducted through a self-administrated structured questionnaire and involved 123 residents and fellows who deal with children in Jeddah, SA. The participants were recruited using convenient sampling methods. Descriptive statistics, t-test, and Chi-square test were used for statistical analysis.

Results

We found that approximately 78% would report their findings to the legal authority, document them, and assess their consistency with parents and the child's explanation. However, only 41.5% of the participants would report CAN to the proper authority. Most participants believed that CAN should be redefined according to Saudi culture and religious standards. In contrast, 68.9% of the participants believed that CAN cases are under-reported in SA. The main barrier to not reporting is the fear of consequences (63.4%). About 77% of the participants agreed to the need for further training. Identifying the CAN indicators was higher among those who handled a CAN case previously (median = 66.67, p = 0.023).

Conclusions

In conclusion, the study showed that appropriate undergraduate and postgraduate curriculum training should be developed to strengthen future healthcare practitioners in dealing with CAN cases to protect children's welfare.

## Introduction

There is a widespread problem of violence that affects all countries and communities in the world [1]. According to the World Health Organization (WHO), “violence against children includes all forms of violence against people under the age of 18 years, whether perpetrated by parents or other caregivers, peers, intimate partners, or strangers” [2]. According to cases of maltreated children under the age of five were presented in Saudi Arabia (SA) in the early 1990s, since then, child maltreatment in SA started to attract the attention of healthcare professionals. A growing number of health professionals are becoming aware of child maltreatment in SA [3]. Moreover, child neglect is estimated to have a local prevalence of 15% [4].

While there are many different forms of interpersonal violence that impact children, one of the most prevalent forms is child abuse and neglect (CAN). According to the WHO, CAN refers to the physical, sexual, and psychological abuse as well as the neglect of newborns, kids, and teenagers by parents, guardians, and other authority figures; these things typically happen at home, but they can also happen in other places like orphanages and schools [2]. CAN can be broadly classified into four categories: physical abuse, psychological abuse, sexual abuse, and neglect. Healthcare personnel, particularly those who work with children and are directly involved in addressing this complex issue, play a crucial role. Since they are frequently the first professionals to deal with the severe end of the spectrum, especially in the early stages of infancy, their role is crucial. They must therefore be knowledgeable about the behavioral and physical symptoms of CAN in both children and caregivers. They should also understand the importance of following the right procedures and, most importantly, informing the appropriate services.

Health professionals need to be skilled in CAN identification and management, but studies have indicated a knowledge gap that may be the result of insufficient training [4]. Simultaneously, further investigation is required to explore healthcare practitioners' attitudes and knowledge about training [5,6]. Thus, in order to develop instructional strategies that centre on improving their skills in the identification and reporting of CAN and acting quickly to potentially save a child's welfare and life, the purpose of this study was to ascertain the knowledge and attitudes regarding CAN of physicians enrolled in training programs in Jeddah, SA.

## Materials and methods

Study design and setting

We performed an electronic cross-sectional survey using convenient sampling technique by distributing to the social media groups of the related departments to the residents and fellows in pediatric and psychiatry hospitals, and those in the ear, nose, and throat (ENT) and family medicine residency training programs in Jeddah, SA during a period of two months (21 August to 22 October 2020) using questions from a validated questionnaire published in a previous study in SA [4] and developed by the research team, and piloted on 30 postgraduate medical doctors who deal with children prior to the beginning of the study. The self-efficacy scale was used to evaluate the convenience of the residents in caring for abused and neglected children, built according to the Guide For Constructing Self-Efficacy Scales [7]. All potential participants were informed in detail about the study aims and data confidentiality. Postgraduate medical doctors who agreed to participate in the study were asked to complete the anonymous online questionnaire regarding their knowledge of CAN. A resident or fellow doctor who deals exclusively with adults is excluded from this study. The questionnaire was written in English and consisted of questions on demographics (age, gender, marital status, training category, number of children, year of residency program, and years of experience) and questions regarding the education they had received from their faculty’s curriculum or elsewhere, and questions on participants' training speciality or workplace. There were five general domains of questions regarding CAN knowledge (physical abuse, emotional abuse, sexual abuse, neglect, and CAN indicators). There are also questions to assess the attitudes toward CAN. A total of 11 answers were given on a five-point Likert scale (“strongly agree”, “agree”, “not sure”, “disagree”, or “strongly disagree”).

Statistical analysis

Statistical analyses were determined using the Statistical Package for the Social Sciences (IBM SPSS Statistics for Windows, IBM Corp., Version 25.0, Armonk, NY). Descriptive statistics were used to summarize the data on demographic characteristics and knowledge and attitudes regarding CAN. The Kruskal-Wallis test and chi-squared test were used to determine statistically significant associations between demographic characteristics and a doctor’s knowledge and attitude. The level of significance was set to 0.05.

## Results

Participants demographics

In total, 123 residents participated in this study, of whom 84 (68.3%) were females and 39 (31.7%) were males. The average age of the participants was 30 years old. The majority of the participants (91.1%) were enrolled in a postgraduate training program, with 44 (35.8%) of the participants enrolled in a pediatric training program. During their training, 101 (82%) of the participants dealt with CAN cases. A total of 48 (39%) participants attended a course or workshop related to CAN. There are 51 (41.5%) participants who would report CAN to the National Family Safety Registry (NFSR) through local child protection teams. It was reported that 68 (55.3%) of participants received some kind of training about CAN during their medical school and/or postgraduate training. On the other hand, 55 (44.7%) did not receive any training regarding CAN (Table 1).

**Table 1:** Baseline characteristics of the participants

Baseline Characteristics	Overall (n=123)
Age	n
Mean	30.1 years
Standard deviation	±4.7 years
Range	24.0-52.0 years
Gender	n (%)
Female	84 (68.3%)
Male	39 (31.7%)
Marital status	
Married	56 (45.5%)
Not married	67 (54.5%)
Training category	
Resident/fellow	112 (91.1%)
General practitioner	11 (8.9%)
Specialty	
Pediatric	44 (35.8%)
General practitioner	47 (38.2%)
Others	32 (26.0%)
Year of residency	
First year	19 (16.8%)
Second year	18 (15.9%)
Third year	23 (20.4%)
≥4 years	35 (31.0%)
Fellow	18 (15.9%)
Years of experience since starting work	
Mean	4.3 years
Standard deviation	±3.5 years
Range	0.0 - 20.0 years
Do you have any children?	
No	81 (65.9%)
Yes	42 (34.1%)
Have you been a victim of child abuse and neglect?	
No	105 (85.4%)
Yes	18 (14.6%)
Friend or relative victim of child abuse or neglect?	
No	59 (48.0%)
Yes	64 (52.0%)
Dealt cases of child abuse and neglect in the medical field?	
No	22 (17.9%)
Yes	101 (82.1%)
Attended courses, workshops, conferences, or awareness campaigns?	
No	75 (61.0%)
Yes	48 (39.0%)
Number of training lectures/courses/workshops received	
0	63 (51.2%)
1	45 (36.6%)
≥2	15 (12.2%)
Where to report child abuse and neglect?	
I do not know	11 (8.9%)
The Ministry of Health	11 (8.9%)
National Family Safety Registry	51 (41.5%)
Police	10 (8.1%)
Social agency	40 (32.5%)
Are you aware of the National Family Safety Registry?	
No	81 (65.9%)
Yes	42 (34.1%)

Participants knowledge

As shown in Table 2, the five main categories of CAN were assessed in terms of knowledge. In general, participants had a higher level of knowledge in regard to physical abuse (median=78.33, *p*=0.019) and child neglect (median=88.57, *p*=0.007) compared to other categories.

**Table 2:** Child abuse and neglect knowledge among residents and fellows and factors affecting their knowledge CAN: Child abuse and neglect. * indicates a statistically significant *p*-value.

Score	Physical abuse	Emotional abuse	Neglect	Sexual abuse	Indicators of CAN
	n (%)				
Poor knowledge	20 (16.3)	38 (30.9)	15 (12.2)	70 (56.9)	27 (22.0)
Adequate knowledge	78 (63.4)	64 (52.0)	39 (31.7)	40 (32.5)	61 (49.6)
Good knowledge	25 (20.3)	21 (17.1)	69 (56.1)	13 (10.6)	35 (28.5)
Gender	Mean scores				
Male	75	74	77.143	66.667	75.385
Female	78.333	76	88.571	70	79.231
U	1249	1393	1123	1329.5	1306
z	-2.120	-1.335	-2.811	-1.686	-1.808
p	0.034*	0.182	0.005*	0.092	0.071
Having children					
No	75	74	82.857	70	75.385
Yes	81.667	80	91.429	66.667	79.231
U	1041	1163.5	1206.5	1387.5	1531.5
z	-3.529	-2.873	-2.649	-1.681	-0.906
p	<0.001*	0.004*	0.008*	0.093	0.365
Dealt with CAN cases					
Yes	78.333	76	88.571	66.667	78.462
No	75	75	84.286	71.667	73.846
U	940	1049	970	913.5	767
z	-1.131	-0.410	-0.935	-1.310	-2.274
p	0.258	0.682	0.350	0.190	0.023*
Attended CAN courses/workshop					
Yes	78.333	79	90	66.667	77.692
No	76.667	74	85.714	70	75.385
U	1405	1513	1578.500	1653.500	1731.500
z	-2.053	-1.491	-1.153	-0.764	-0.356
p	0.040*	0.136	0.249	0.445	0.722
Training category					
Resident/Fellow	78.333	76	88.571	68.333	76.923
GP	68.333	70	62.857	66.667	63.077
U	351.500	411.500	313	563.500	481
z	-2.350	-1.817	-2.697	-0.468	-1.199
p	0.019*	0.069	0.007*	0.640	0.231
Level of training					
First year	46.211	50.842	46.079	60.868	58.947
Second year	59.528	57.056	56.444	58.972	59.722
Third year	51.174	57.391	51.848	64.348	53.196
Fourth year and above	56.686	48.757	54.571	48.657	51.557
Fellow	73.917	78.972	80.389	57.778	67.667
H	7.741	11.043	12.151	3.807	3.388
p	0.102	0.026*	0.016*	0.433	0.495
Training specialty					
General practitioners	55.149	52.543	56.872	49.074	55.277
Paediatrics	69.148	66.705	65.875	67.057	75.523
Others	62.234	69.422	64.203	74.031	53.281
H	3.524	5.486	1.629	10.820	9.951
p	0.172	0.064	0.443	0.004*	0.007*

Knowledge and sociodemographic association

In terms of physical abuse and neglect, female participants scored higher (median=78.33, *p*=0.034) than male participants (median=88.57, *p*=0.005). Compared to participants without children, those who had children scored higher on a scale measuring knowledge of physical abuse (median=81.67, *p*<0.001), emotional abuse (median=80.00, *p*<0.004), and neglect (median=91.43, *p*<0.008).

Knowledge and CAN experience

Participants who previously handled CAN cases scored higher on the indicators (median=66.67, *p*=0.023). A significant difference was not observed in any other category of CAN knowledge. However, there were higher knowledge scores regarding physical abuse among participants who attended a CAN workshop or course (median=78.33, *p*=0.04). Otherwise, there was no significant difference noted regarding other CAN knowledge categories.

Knowledge and training program

Pediatric residents have the highest median score for sexual abuse (median=70.00), followed by residents in other specialities (median=70.00) and general practitioners (median=63.33). According to a Kruskal-Wallis test, the difference was statistically significant, H=10.82, *p*=0.004, ηH=0.07. Participants who were in a fellowship program had higher knowledge scores in emotional abuse (median=81.00, *p*=0.026) and neglect (median=91.43, *p*=0.016) than others. Paediatricians had the highest median value for the indicators for CAN score (86.92), followed by general practitioners (75.38) and others (75.38). There was a statistically significant difference, based on the Kruskal-Wallis test: H=9.95, *p*=0.007, ηH=0.07.

Participants perception

Table 3 shows the general perception of CAN. Thirty-four (28.6%) participants received adequate training regarding CAN. As for 78 (63.4%) participants, they believe that CAN should be redefined based on Saudi cultural and religious standards. Twenty-four (19.5%) of participants deemed the current supportive services provided by the CAN adequate. There were 52 (42.3%) participants who were aware of their hospital's CAN reporting system. Most participants, 94 (76.4%), are willing to report suspected cases of CAN. Note that 17 (13.8%) participants prefer to report life-threatening CAN injuries. Eighty-six (69.9%) participants believe that CAN cases in SA are underreported.

**Table 3:** General perceptions of the training physician about child abuse and neglect

Statements	Strongly disagree	Disagree	Not sure	Agree	Strongly agree
	n (%)				
1. I have adequate training to deal with child abuse and neglect	15 (12.2%)	45 (37.4%)	28 (22.8%)	26 (21.1%)	8 (6.5%)
2. We need to redefine child abuse and neglect in Saudi Arabia according to our culture and religion	10 (8.1%)	17 (13.8%)	18 (14.6%)	55 (44.7%)	23 (18.7%)
3. The present supportive services to deal with child abuse and neglect in Saudi Arabia are adequate	21 (17.1%)	29 (23.6%)	49 (39.8%)	21 (17.1%)	3 (2.4%)
4. I prefer resolving a case of child abuse and neglect on my own rather than reporting it	52 (42.3%)	26 (21.1%)	29 (23.6%)	10 (8.1%)	6 (4.9%)
5. I am aware of the reporting pathway of child abuse and neglect in my hospital	13 (10.6%)	31 (25.2%)	27 (22.0%)	46 (37.4%)	6 (4.9%)
6. I am willing to report all suspected cases of child abuse and neglect	1 (0.8%)	7 (5.7%)	21 (17.1%)	53 (43.1%)	41 (33.3%)
7. I prefer limiting my reporting of child abuse and neglect to those that are life-threatening	47 (38.2%)	33 (26.8%)	26 (21.1%)	15 (12.2%)	2 (1.6%)
8. Child abuse and neglect are underreported in Saudi Arabia	4 (3.3%)	7 (5.7%)	26 (21.1%)	39 (31.7%)	47 (38.2%)

Participants attitude

Table 4 shows the findings regarding the attitudes of the participants toward the abuse and neglect of children. Approximately 78% of participants plan to report their findings to the authorities, and 73.2% would document their findings. Of the respondents, 70% of them would assess the consistency of the parents and the child's explanations of the clinical findings they observed. In comparison, 52.8% would ask the child's parents about any signs/symptoms noticed during the clinical assessment that were likely indicative of CAN. Also, Table 4 shows findings regarding the barriers to reporting CAN. In this study, 63.4% of participants expressed concerns about possible violence or negative consequences for the child. Of the respondents, 54.5% were unaware of the reporting procedure, and 34% did not believe there is a legal obligation to report abuse and neglect of children. Also, 59.3% feared aggressive responses from the child's family or parents, and 44.7% believe that reporting CAN in our community has not yet become acceptable.

**Table 4:** Training physician attitudes and barriers toward reporting child abuse and neglect

The factor	Answers	n (%)
Attitude	Ask the child and parents about the signs/symptoms you notice	65 (52.80%)
	Document the signs/symptoms and your suspicion on file	90 (73.20%)
	Monitor the case during the following visits	54 (43.90%)
	Report to legal authority	96 (78.00%)
	Check the consistency of parents’ and child's explanations with findings	86 (69.90%)
	Do nothing	1 (0.80%)
	I do not know	4 (3.30%)
Barriers	Reporting procedure is unclear	67 (54.50%)
	Reporting child abuse or neglect to authorities is not yet acceptable in our community	55 (44.70%)
	Fear of violence or unfavorable consequences to the child	78 (63.40%)
	Fear of aggressive response from the child’s family or parents	73 (59.30%)
	There is no legal obligation to report child abuse and neglect	42 (34.10%)
	Uncertainty about the diagnosis of child abuse or neglect	40 (32.50%)

## Discussion

Abuse against children was not properly acknowledged and reported in SA as early as the 1990s [8]. This was followed by a number of cases that were later identified and led to the creation of the national team, whose task was to identify and assess suspected cases of CAN [9]. By law, all healthcare providers are required to report instances of child abuse. Reporting CAN, however, has never been easy [10]. A law prohibiting CAN was put into effect in SA between the end of 2013 and the end of 2014, safeguarding children's rights and promising improvements in the treatment of this problem. By law, all healthcare providers are required to report instances of child abuse. Reporting CAN, however, has never been easy. SA passed laws in August 2013 and November 2014 to prevent CAN and to uphold the rights of children, which was anticipated to enhance the treatment of this problem in the medical field [2].

CAN in SA from a trainee perspective

By measuring participants' perceptions of CAN, it was discovered that most of them (69%) thought CAN existed in SA. This result aligns with a study conducted in SA by Alanazi et al., wherein 87% of postgraduate medical doctor providers stated that CAN is present in the country [2]. Conversely, it is widely acknowledged that culture and ethnicity have an impact on CAN [2]. Similar to the findings of Aldukhayel et al., where most of their participants believed in the importance of redefining CAN in accordance with cultural and religious traditions [11], the majority of respondents (63%) in the current study felt that the concept of CAN should be redefined in accordance with Saudi culture and religious obligations.

CAN reporting

Previous research in SA has demonstrated a markedly low level of CAN reporting knowledge [12]. According to Habib et al., 66-79% of the paediatricians who took part in their research understood what CAN reporting was all about [13]. Eighty-seven per cent of participants in the current study were familiar with the procedures for reporting to the legal authority in SA, and 73% were aware of the reporting sites, which is higher than these previous reports. Furthermore, the results of this study are consistent with those of another study carried out in Dammam, SA, which revealed that over 90% of paediatricians were aware of the reporting sites [14].

When the barriers to reporting cases of CAN were evaluated, the majority of participants in this study felt that the main reason was to prevent potential violence or unfavourable consequences for the child (60%); 59% said it was because they were afraid of the child's family or parents reacting aggressively; 54.5% said they were unaware of the reporting process; 44.7% said that reporting CAN is not yet accepted in our community; and 34% said there is no legal requirement to report CAN. According to a different study, the primary reason was a lack of trust in social service providers [2].

CAN knowledge affected by what?

It has been demonstrated that the training programs raise awareness and CAN recognition skills, which eventually leads to an increase in the frequency of reported instances [2]. However, 39% of the participants in our study attended a course or workshop about CAN, and 55.3% of participants received some training about CAN throughout their medical school and/or postgraduate training resulting in a statistically significant superiority in knowledge (*p*<0.05). As a result of attending a CAN workshop or training program, participants scored higher on one of the child abuse categories, namely physical abuse (median=78.33, *p*=0.04). Even though participants in a fellowship program had higher knowledge scores regarding emotional abuse (median=81.00, *p*=0.026) and neglect (median=91.43, *p*=0.016), this might conclude that the focus of courses and workshops is more on physical abuse than the other categories. Thus, it needs reinforcement and revision. However, finding that physical abuse is the most recognized type is unlike another Saudi study that found sexual abuse is the most recognized type [5]. However, one limitation in the interpretation of the study findings is the nature of the included sample which was restricted to undergraduates.

Regarding the main categories that assess the knowledge of CAN, we found that females are more likely to recognize physical abuse (median=78.33, *p*=0.034) and neglect (median=88.57, *p*=0.005). Females show a higher level of knowledge regarding physical abuse in Merwass et al.'s study and regarding neglect in Alnasser et al.'s study [5,12]. Also, knowledge of physical (median=81.67, *p*=<0.001) and emotional (median=80.00, *p*=<0.004) abuse, and neglect (median=91.43, *p*=<0.008) all had statistically significant associations among participants who had children compared to those without children. Married paediatricians in Saudi studies show a higher level of knowledge regarding CAN [13]. Moreover, there were statistically significant associations between knowing the CAN indicators and having previously dealt with a CAN case (median=66.67, *p*=0.023). On the other hand, having experience dealing with CAN does not make a significant difference in the other knowledge categories. Experience played a role in identifying indicators and neglect in another Saudi study [5]. Consider that the study pooled all who have experience together, including consultants, unlike our study, which includes only under-training physicians.

Furthermore, another study acknowledged the impact of culture and religion on how people perceive and comprehend CAN [2]. SA offers a special framework for addressing CAN because of its unique cultural and religious traditions. The need for cultural sensitivity in addressing this issue is highlighted by the finding that most study participants supported redefining the definition of CAN in accordance with Saudi cultural norms and religious obligations [11]. Also, according to the current study, women were found to be more capable than men of identifying physical abuse and neglect [5,12]. This result is in line with earlier Saudi Arabian research projects [5,12]. Furthermore, individuals with children showed higher knowledge scores in a number of categories related to CAN, indicating that firsthand experiences as parents may help people become more aware of and comprehend these problems [4].

According to the current study, it is legally required for all healthcare providers in SA to report any suspected and confirmed cases of CAN [2]. Numerous nations have enacted laws requiring reporting in order to guarantee the detection and handling of child abuse cases. These regulations seek to safeguard minors and offer a structure for medical professionals to carry out their duties of disclosing possible instances [15]. Healthcare professionals frequently encounter difficulties and moral conundrums when determining whether to report instances of CAN, even in spite of the legal requirement [10]. In cases of CAN, law enforcement, social workers, healthcare providers, and other pertinent professionals must work together in a multidisciplinary manner. Although the current study concentrated on the viewpoint of medical residents, it is imperative to stress that interdisciplinary collaboration is necessary to effectively address CAN [16]. The identification, reporting, and delivery of suitable interventions for victims of child abuse can all be improved by such collaboration [17]. Even though the current study showed that training programs and workshops had a positive effect on participants' knowledge of CAN, it is important to stress that further education and training are still necessary. Healthcare professionals should keep up to date on the most recent findings, recommendations, and best practices for recognizing and handling CAN cases because these cases are constantly changing [18]. The confidence and skill of healthcare professionals in identifying and handling CAN can be enhanced with ongoing education [19].

Nonetheless, in order to prevent CAN, it is imperative that public awareness be raised. Public awareness campaigns can aid in educating communities about risk factors, available support services, and the different types of CAN [20,21]. One of the barriers to reporting CAN, as mentioned in the current study, is the lack of social acceptance of doing so [2,22]. Creating a supportive environment for reporting and addressing cases of CAN can be facilitated by raising awareness and altering societal attitudes.

Limitations

The cross-sectional study design may present a potential selection bias and unequal samples across different medical specialities in the study. These limitations were addressed by increasing the sample size.

## Conclusions

In conclusion, our study outcomes represented the majority of participants who were aware of their future responsibility to protect vulnerable children and report suspected cases of CAN; at the same time, they were willing to obtain further education. It is recommended that undergraduate curricula emphasize marriage counselling and appropriate training to strengthen and increase future healthcare professionals' confidence in handling suspected cases of CAN and safeguard the welfare of children. For future studies, researchers should explore the advantages of marriage and starting a family and its potential role in enhancing CAN reporting and detection.
